# DYNASTI—Dynamic Multiple RPL Instances for Multiple IoT Applications in Smart City

**DOI:** 10.3390/s20113130

**Published:** 2020-06-01

**Authors:** Sidnei Junior, André Riker, Bruno Silvestre, Waldir Moreira, Antonio Oliveira-Jr, Vinicius Borges

**Affiliations:** 1 Institute of Informatics (INF)—Federal University of Goiás (UFG), Goiânia 74690-900, Brazil; sidneijunior@usp.br (S.J.); brunoos@inf.ufg.br (B.S.); antoniojr@ufg.br (A.O.-J.); 2 Institute of Exact and Natural Sciences (ICEN)— Federal University of Pará, Belém 66075-110, Brazil; afr@ufpa.br; 3 Fraunhofer Portugal AICOS, 4200-135 Porto, Portugal; waldir.junior@fraunhofer.pt (W.M.);

**Keywords:** smart city, Internet of Things, LLN, RPL, multiple instances and applications

## Abstract

Internet of Things (IoT) is evolving to multi-application scenarios in smart cities, which demand specific traffic patterns and requirements. Multi-applications share resources from a single multi-hop wireless networks, where smart devices collaborate to send collected data over a Low-Power and Lossy Networks (LLNs). Routing Protocol for LLNs (RPL) emerged as a routing protocol to be used in IoT scenarios where the devices have limited resources. Instances are RPL mechanisms that play a key role in order to support the IoT scenarios with multiple applications, but it is not standardized yet. Although there are related works proposing multiple instances in RPL on the same IoT network, those works still have limitations to support multiple applications. For instance, there is a lack of flexibility and dynamism in management of multiple instances and service differentiation for applications. In this context, the goal of this work is to develop a solution called DYNAmic multiple RPL instanceS for multiple ioT applicatIons (DYNASTI), which provides more dynamism and flexibility by managing multiple instances of RPL. As a result of this, the traffic performance of multiple applications is enhanced through the routing, taking into consideration the distinct requirements of the applications. In addition, DYNASTI enables the support of sporadic applications as well as the coexistence between regular and sporadic applications. DYNASTI achieved results that demonstrate a significant improvement in reducing the number of control messages, which resulted in increased packet received, decreased end-to-end delay, reduced energy consumption, and an improvement in service differentiation to multiple applications.

## 1. Introduction

The recent emerging advances on the Internet of Things (IoT) [1,2] can be easily found several smart mobile and fixed devices (e.g., sensors, cameras, 3D glasses, actuators, and several others). When the resources of these smart devices are combined each other, they offer new monitoring and control capabilities which are able to perform intelligent activities [3,4].

There are a lot of IoT wireless technologies and protocols (e.g., Long Range (LoRa)/LoRaWAN, NarrowBand-IoT (NB-IoT), LTE-M, SigFox, ZigBee, and among others [5,6,7]) that can be applied to provide communication for the smart devices. For instance, a very actual and interesting solution is the LoRaWAN Class B that can be applied for the coordination of distributed interface protection systems in smart grids [8].

In this context, several new application scenarios and networks arise, such as autonomous controlling in smart grid applications [9,10], smart parking management [11], and smart campus which is a small scale sample of smart cities. For instance, Neighborhood Area Network (NAN) in smart grid enables power distribution with the ability of monitoring and controlling electricity delivery in specific areas. Most of these scenarios have multiple applications deployed in the network [9,12,13].

These multiple applications have different traffic patterns (e.g., regular, sporadic, different sending intervals between messages, etc.); the regular applications that communicate constantly throughout the network lifetime and, sporadic applications that demand communication for a short period of time and usually have critical requirements [14]. In addition, both types of traffic pattern (sporadic and regular) can have normal or critical requirements [15]. Furthermore, they can have distinct types of requirements (e.g., reliability, delay, flow, losses) and different parameters of these requirements (e.g., maximum delay of 1 s, minimum delay of 150 ms) [16]. Moreover, these heterogeneous applications require sharing resources at the same in the network [17].

The smart devices have limited memory, processing power, and reduced battery life which makes IoT scenarios a Low-Power and Lossy Networks (LLNs) [18]. Thus, the Routing over Low-Power and Lossy Networks Working Group (RoLL WG) addresses IoT LLN scenarios which provide solutions that enable communication between smart devices. These devices are connected to each other through multi-hop wireless communication to gather information for multi-applications [19].

RoLL WG standardized the Routing Protocol for Low Power and Lossy Networks (RPL) [20] in which the concept of instances emerged as an alternative solution for dealing with the specificities of multiple applications that coexist in LLNs. Instances virtually divide the network in multiple logical subdivisions, enabling that multiple applications with different classes of Quality of Service (QoS) coexist at the network layer [21,22]. Each instance can be individually configured to deal with the QoS requirements of a specific kind of traffic. The RFC6550 [20] mentions the possibility of deploying multiple RPL instances. These instances can provide flexibility in routing and enable QoS for multiple applications that coexist in the same scenario.

It is important to note that RoLL WG did not specify how to implement multiple instances. For this reason, there is investigation about the development of multiple instances for RPL [21,22,23,24,25,26]. However, the proposals present limitations such as:

- Limiting a single instance per application and no flexibility of instances management. As a result of this, the instances are statics. In other words, it means that each application is associated with a single routing metric and a single interval between application messages throughout the application lifetime on the network, since the applications are associated with a single instance. If the applications could associate to multiple instances, this flexibility would improve the service differentiation for each kind of applications, since each application could be adapted its traffic class.
- Sporadic applications that eventually send messages are not supported. It is important to emphasise that the RPL control and applications messages are currently generated and sent at regular intervals throughout the lifetime of the network. The related work implementations do not reflect a network with different traffic patterns.
- Multiple instances performing occurs alternately: one single instance each time. When one instance is communicating, the remainder instances are in wait mode in a First In First Out (FIFO) queue. Thus, there is no way to prioritize instances. This aspect makes very difficult to support applications with different levels of critical requirements such as delay.

This article proposes DYNAmic multiple RPL instanceS for multiple ioT applicatIons (DYNASTI), a solution that extends the implementation of multiple instances in RPL, in order to provide more dynamicity and flexibility to routing, thus more optimizing QoS and traffic performance. This solution allows that multiple IoT applications with different requirements communicate in the same LLN. Thus, the DYNASTI enables:

- Support to sporadic applications.
- Implementation of concurrency between multiple instances.
- Providing more dynamic and flexible applications, allowing each application to have different instances over time.
- Adapting to more critical and sporadic applications that eventually can run on the network (service differentiation between applications with critical requirements).
- Interruption and adjustment of control and application message transmissions.

We implemented DYNASTI on Contiki Operating System (Contiki-OS Version 3.0) [27] and evaluated it using COntiki OS JAva (COOJA) simulator [28].

To the best our knowledge to date, there is no work in the literature addressing the shortcomings of existing multi-instance developments allowing them to be performed with multiple applications with different traffic requirements and patterns on the same LLNs through a management dynamic and flexible of instances.

This paper is structured as follows. Section 2 includes the main concepts and definitions of RPL protocol. Section 3 describes the main related work focusing on proposals of the multiple RPL instances in the literature. Section 4 presents all the developed mechanisms in the DYNASTI, while Section 5 discusses the the assessment of the proposal through a performance evaluation based on simulation. Conclusions and future work are presented in Section 6.

## 2. RPL Background

RPL is a distance vector routing protocol based on Internet Protocol Version 6 (IPv6) and provides multipoint-to-point/ point-to-multipoint communications for LLNs [29]. RPL distinguishes upward and downward traffic according to the direction that messages are transmitted. For RPL, downward indicates that the communication is going from the root to the nodes, and upward means that information is flowing from the nodes to the root. The node called *sink* or root is the central point and most of the times it acts as gateway. RPL creates and maintains a tree topology, named Destination Oriented Directed Acyclic Graph (DODAG), rooted at the gateway.

Each node has a rank based on hop-distance from the root in a DODAG, which increases by going down the tree from the root. In general, it reveals the node’s individual position relative to other nodes taking the root as reference. The exact calculation of the rank is specified by an objective function (routing metric), which can consider other aspects, such as delay, energy consumption, or path loss [30,31].

RPL is designed to allow the system to create and run more than one RPL instance. Multiple instances of RPL can coexist in the network and multiple DODAGs can be constructed in each instance, but a node belongs to a single DODAG for each RPL instance it joined. The idea of multiple RPL instances comes from the need of optimizing the network paths for different objective functions. The same network nodes can run two or more RPL instances to serve a performance criterion, given by an objective function. For instance, one RPL instance seeks to reduce the latency while another instance aims to reduce the energy consumption [22].

The DODAGs construction process is distributed and initiates with the announcement of a new DODAG, to which the nodes can join. When a new node joins the new DODAG, it must provide at least one parent node as a default route towards the root. A parent of a node is defined as one of the immediate successors of the node on a path towards the root. RPL exchanges frequently three types of control messages to find the parent towards the root and also to enable the communication between the root to the nodes. DODAG Information Object (DIO) message contains information that makes a discovered of a DODAG. This message carries the RPL instance ID, DODAG ID, Mode of Operation, Rank of the sending node, Metric Container, Routing Information, and DODAG Configuration. DODAG Information Solicitation (DIS) messages are sent by nodes who have not joined any DODAG. The message is sent to neighboring nodes to discover the DODAGs nearby. The DIS message is answered by the nodes via a DIO message. Destination Advertisement Object (DAO) message is used to propagate destination information upward along the DODAG.

A node may also use DIS to probe its neighborhood in order to receive a DIO message and find new available DODAGs. After receiving the DIO message, a node can join a DODAG tree. Nodes will receive and process DIO messages from many nodes and make a decision to join the graph based on the objective function and local policies. For this reason, DIO messages carry more information than other RPL messages. The DAO message also propagates RPL routing information, but it contains upward route information which is valid only to members of the same DODAG. RPL uses the trickle algorithm to manage the DIO messages rate. On the one hand, if there is a smaller number of DIO messages sent than a specific threshold within the interval, RPL reduces the trickle timer value. On the other hand, if there is a greater number of DIO messages than a specific threshold, the trickle interval (DIO message rate sending) is doubled up to a maximum value.

## 3. Related Work

We describe the main research work addressing the use of multiple RPL instances in this section. Initially, the use of multiple RPL instances enhanced flexibility and heterogeneity for IoT networks through the use of one or more objective functions, so that each instance could use a distinct objective function (routing metric).

Long et al. [23] proposed the first work to address a multiple RPL instances, providing high Packet Delivery Rate (PDR) and low latency. Rajalingham et al. [24] developed a QoS engine considering smart grid requirements and multiple instances, but both aforementioned research work was limited to using only one instance per application.

Banh et al. [25] proposed using instances in a two-application environment, using the Hop Count (HC) metric for regular application and the Expected Transmission Count (ETX) metric for critical application in which each metric was associated with an instance. To evaluate the work, the authors analyzed the convergence time for network construction by measuring the time difference between the first DIO message sent by the sensor nodes and the last DIO message associated with the DODAG. Latency and rate of incoming packets were evaluated, but no differentiation in traffic patterns for IoT networks was addressed.

Barcelo et al. [26] proposed a cooperative strategy based on the RPL protocol (called C-RPL), where nodes are defined according to C-RPL instances. The strategy considers the trade-off between the performance and energy consumption associated with each coalition. In their work, a coalition concerns one or more collaborating instances, sharing their nodes dynamically to improve quality of service for multiple applications. In other words, nodes migrate from one instance to another in order to balance the network to achieve better quality of service for applications.

Node coalition can support network traffic load balancing and improve network life when some nodes are low in power. However, the same reasoning may not be valid for link quality, which is an aspect to consider in critical applications. Moreover, it is not always possible to minimize interference by switching nodes between instances, as the nodes themselves may be the cause of such network interference or interference may be caused by external sources that cannot be handled or controlled. In addition, the node coalition, when considering interference, can generate instabilities due to the intermittence of this phenomenon. Each application is associated with a single instance in C-RPL, and each instance is associated with a single routing metric to meet application requirements. In addition, C-RPL employs a centralized network approach, where a node has a global network view to initiate the coalition process, but RPL works locally and distributed, where nodes only have information from their adjacent neighbors. Furthermore, the work was evaluated in MATLAB^®^ using a slotted listening duty cycling scheme, which is a simple implementation of the physical and Medium Access Control (MAC) layers which affects the network lifetime, delay, and congestion level.

The most recent multiple instance proposal for RPL is addressed by Nassar et al. [21,22]. The research work presents a model in which multiple applications coexist on the same infrastructure and uses a multi-purpose metric to differentiate QoS, demonstrating that quality improvement can be achieved by using two applications on a network, through the RPL protocol. The authors proposed a new objective function called Objective Function for Quality of Service (OFQS) that contains the multipurpose routing metric mOFQS, which uses delay, link quality, and battery state of nodes across three levels (full, intermediate, and low). Thus, each instance has a distinct mOFQS metric, prioritizing different requirements according to the variation of the alpha and beta parameters, which may vary as follows: alpha=1−beta,0<alpha<1 and 0<beta<1. In this sense, if the value of alpha is prioritized, the metric chooses routes through link quality and delay information. On the other hand, if beta is the priority, then the metric behaves considering node battery levels.

However, communication on the network occurs in an orderly manner, i.e., each application is associated with a single instance on a static basis and employs a kind of queue for transmission: the first application in the queue transmits all its application messages and goes to the end of the queue, turning to the next application. RPL control messages do not follow this rule, all instances continue to send these control messages freely. This procedure occurs throughout the network’s lifetime, statically and without any interruption of instance control messages that are not transmitting messages. Thus, this approach can lead to unnecessary overhead as application messages are transmitted alternately.

However, the solutions presented by the authors support differentiation service for applications with different requirements through the association of routing metrics and instances. The solution does not support sporadic applications that appear on the network eventually with critical low delay or high reliability requirements in lost packets. It is important to stress out that the support for sporadic applications could enable them to send messages on the network more quickly, and there is no need to wait for other applications to terminate their transmissions by following the transmission queue order. Therefore, the approach in [21,22] does not allow regular and sporadic instances to coexist by sharing network resources differently.

Table 1 provides a comparison between DYNASTI and the related work addressing the use of multiple instances and the RPL routing protocol. The comparison is made through the following fields:

- Metric: Which metrics were used to generate the results.
- Number of instances per application: Number of instances per application.
- Service differentiation for applications: Modified instance settings, for instance routing metric, to prioritize applications.
- Traffic Pattern: Existence of sending messages in different patterns.

Approaches of multiple instances by [21,22,23,24,25,26] have some limitations. These approaches employ only one instance per application and there is no instance management, which results in static instances. An application is associated, for example, with a single routing metric and a single interval between application messages at all times.

In addition, implementations do not support sporadic applications that send occasional messages. Importantly, RPL control messages and application messages are generated and sent at intervals throughout the lifetime of the network. Thus, the above approaches do not reflect a network with different traffic patterns.

## 4. DYNAmic Multiple RPL Instances for Multiple IoT ApplicatIons (DYNASTI)

The DYNASTI proposal is a solution that allows more dynamism and service differentiation to support multiple IoT applications with different traffic patterns. DYNASTI makes it possible to manage multiple RPL instances based on the scheduling of application instances to carry out traffic adaptation, accommodating the emergence of sporadic applications. Thus, it is intended to meet the different performance requirements of multiple IoT applications that are using the same LLNs.

### 4.1. Concepts and Definitions: Applications, Traffic Classes, and RPL Instances

The communication of different types of traffic occurs concurrently in IoT scenarios with multiple applications, and each application has different requirements and disputes the resources available on the network. This demands a differentiation of traffic, resulting in a range of scenarios, which motivates the study of these multiple applications and how much their requirements can be supported differently even when computational and network resources are scarce in LLNs.

**Figure 1:**
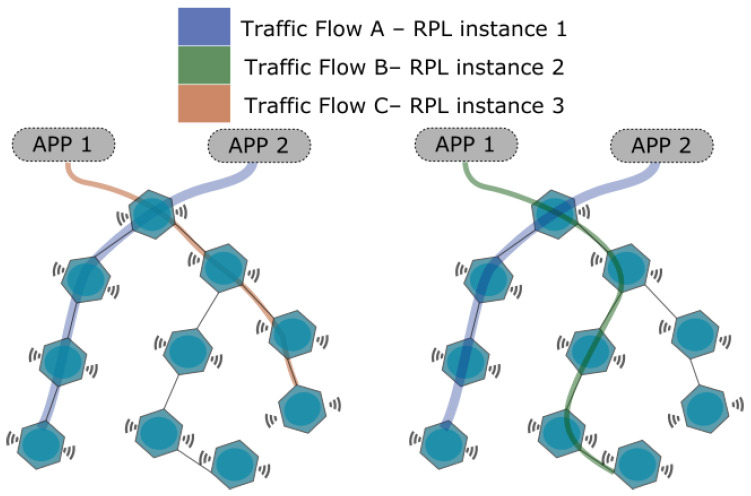
Diagram of the linkage between applications, instances, and traffic classes.

Each application can have more than one instance option in our proposal; however, an active application (running) is associated with a single instance in an instant of time. The instances are employed selectively, so that there is adaptation to the emergence of new applications with requirements of different priorities. To enable this adaptation, we use traffic classes, which are composed of a routing metric and a message sending interval for each instance. Figure 1 illustrates a scenario with multiple applications, where there are three traffic flows. The communication of each flow is supported by different instances of the RPL and therefore different classes of traffic. This allows each stream to have a particular routing metric and transmission interval. For example, application 1 has flows B and C associated with instances 2 and 3, respectively. Consequently, application 1 has two classes of traffic at different times.

**Figure 2:**
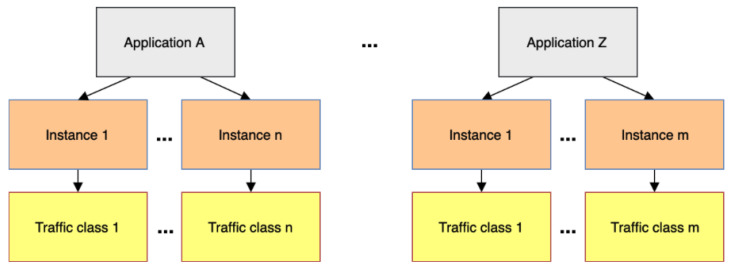
Relationship diagram between applications, instances, and traffic classes.

Figure 2 demonstrates the relationship between application, instance, and traffic class. Each application can have more than one instance, and, for each instance, there is a class of traffic. These classes have different configurations to meet the requirements of critical applications that may appear sporadically.

Each instance of the application employs a distinct metric that allows applications to be more flexible: if the network infrastructure lacks resources, the application can switch from the current instance to another with more appropriate metrics and message sending intervals. This change directly impacts decision-making for the choice of routes and thus frees up resources to meet the demand of other competing applications with critical traffic. The applications, therefore, are not permanently associated with a single class of traffic, making them more dynamic, since they can adapt to coexist with traffic that has more priority.

### 4.2. DYNASTI Architecture

**Figure 3:**
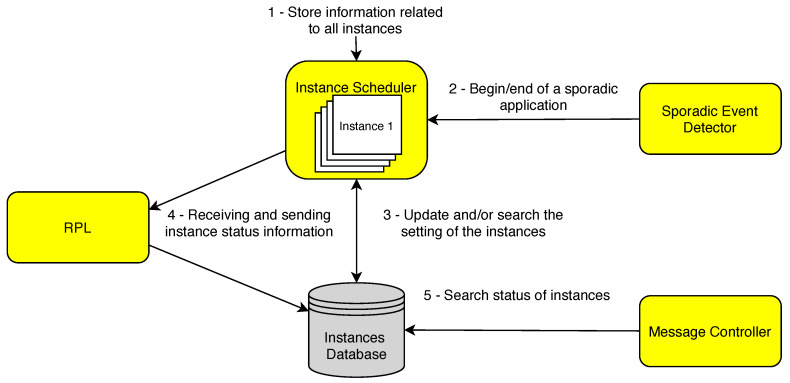
Flowchart of the mechanisms of the DYNASTI solution in the sensor nodes.

DYNASTI aims to schedule RPL instances to modify the traffic class of applications. Instances are activated and deactivated to accommodate, for example, the appearance of a new sporadic application, which also affects the instances of applications that are already running. The architecture of the DYNASTI solution is shown in Figure 3 and is composed of the following parts:

- Instance Scheduler.
- Sporadic Event Detector.
- Instances Database.
- Message Controller.
- RPL.

Initially, the settings of the instances (from regular and sporadic applications) are stored in the Instance Scheduler (step 1). The Sporadic Event Detector is responsible for monitoring the appearance or termination of sporadic applications, generating events for the Scheduler (step 2).

The Instance Scheduler, after receiving the event from step 2, must select which instances to activate or deactivate—in Section 4.3, we detail the strategies adopted by the Scheduler. Then, the status of the instances is updated in the database (step 3) and a notification is sent to the *sink* node describing the instance changes (step 4) so that it is disseminated to the other nodes—this process is further described in Section 4.4.

Finally, the Message Controller queries the *status* of the instances in the database and filters the application and control messages of the disabled instances (step 5). Thus, the Controller is the effective component to perform the deactivation of the instances, preventing any message from being sent on the network, which saves resources.

It is worth mentioning that the sink does not have the Event Detector component, the role of the *sink* in the architecture is to assist in the network initialization (creating the DODAGs, initial status of the instances) and disseminating the information for performing the scheduling of instances in the network.

### 4.3. Instance Scheduler

The Instance Scheduler plays an important role in the DYNASTI solution, as it enables support for sporadic applications while offering service differentiation for applications with different configurations (traffic pattern and requirements). It also has the traffic classes of the instances associated with the applications, such as routing metrics and send interval of the application messages.

The Scheduler has a predefined set of scheduling of active and disabled instances in order to be able to change the instances, thus to support sporadic applications and their coexistence with regular applications. The scheduling of instances happens when a sporadic application is started, forcing the instance to be activated for that application.

**Figure 4:**
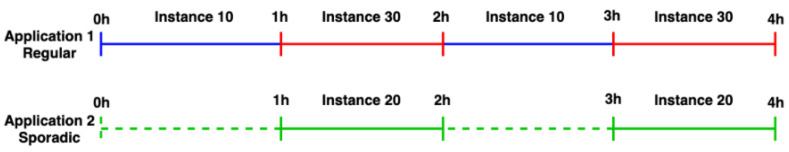
Example of four hours of operation of three schedules with two instances associated with the regular application and one critical application with one instance.

Figure 4 presents a possible scheduling of instances for the scenario of the two applications. Application 1 has two colors for its timeline: the blue color depicts Instance 10 with a traffic class using, for example, the ETX routing metric and a shorter and original interval for sending application messages. The red line reflects Instance 30 of Application 1, using a greater interval for sending application messages than Instance 10 (blue). In this way, Application 1 gives Application 2 more resources.

In this case, the scheduling defines that (i) when Application 1 is running in isolation, it will use Instance 10 and (ii) when Applications 1 and 2 are running concurrently, Application 1 will use Instance 30 and Application 2 will use Instance 20. Initially, Instance 10 is activated during the interval from 0 h to 1 h. In 1 h, Application 2 starts, causing the Scheduler to change the status of Instance 10 to deactivated as well as Instances 20 and 30 to activated. This is maintained until time 2 h when the Scheduler changes the status to activated for Instance 10 and deactivated for Instances 20 and 30, as Application 2 is interrupted. This schedule is also made in the interval of 3 h to 4 h.

It is worth remembering that the role of the Scheduler is to identify which instances should be active or not. The Message Controller component is responsible for suppressing or activating the application and control messages of the instances, disabling, or enabling them.

Thus, when there is a demand for more critical sporadic application on the network, regular less critical applications may employ instances with less stringent traffic classes in order to free up resources for the more critical application. In addition, less critical regular applications do not need to interrupt totally the sending of application and control messages, seeking, whenever possible, to use different routes from the most critical applications based on the routing metric of the traffic class of that instance. After the most critical application ends, less critical applications that had switched instances and, consequently, traffic class, can return to their previous instances, that is, return to their previous traffic class and return to using the resources released by the more critical application.

### 4.4. Dissemination and Storing Information of RPL Instance Scheduling

After the instances are scheduled due to the detection of a sporadic application, the status of the scheduled instances must be updated in the local database of the sensor node that detected the event. The next step is to disseminate these status updates across the network. This information must be propagated to the other nodes of the network so that they can change the instances information. This spread of information scheduling depends on the *sink* node, as the other nodes have a restricted view of the network in RPL. When a node’s scheduler detects the need for update, it uses DAO messages to update the *sink*, informing what the new status of each instance is. Upon receiving the DAO message, *sink* employs the DIO messages to disseminate the status of the instances to the entire network.

DYNASTI defines three types of status for the instances, two of which were previously described as transmission (enabled) and interruption of sending (disabled) instance data, and the third type that is used in the network initialization phase. Each type of status controls the sending of application and control messages required for each phase, as listed below:

- *Status* 1: Instance communicates only control messages.
- *Status* 2: Instance communicates both control and application messages.
- *Status* 3: Instance is deactivated, it does not communicate control or application messages.

**Figure 5:**
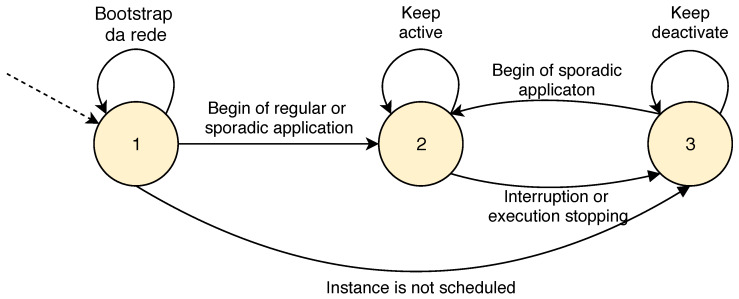
State diagram for instances status.

The state diagram is presented in Figure 5 for sporadic and regular applications demonstrating the status transition. At first, the instance enters in *status* 1 during the network boot period (*bootstrap*) for all predefined instances. The initialization phase aims to create the DODAGs for each instance, assembling the network graph.

Then, the instance is changed to *status* 2, if a sporadic or regular application is started and associated with an instance, when the nodes communicate control and application messages. After, if there is an interruption of the instance due to the emerging of another sporadic application or, if the sporadic application has stopped running, the instance is changed to *status* 3 and remains in this status until it is selected by the Scheduler and returns to *status* 2. Alternatively, instances that have not yet been scheduled can do the transition from *status* 1 to *status* 3.

**Figure 6:**
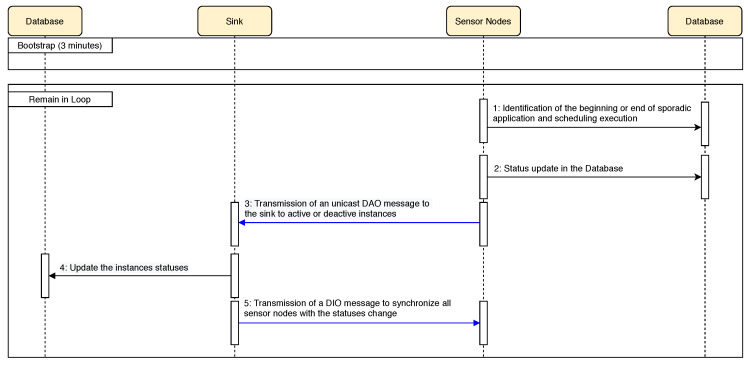
Sequence diagram that shows the interaction between sink, database, and sensor nodes.

The sequence diagram is presented in Figure 6 with the functions that are responsible for disseminating the status of the instances and thus allowing flexible management of the instances. In the initial phase, a network initialization period (*bootstrap*) is performed. This period is required to share instance information between the *sink* and the sensor nodes on the network.

The closed loop (*loop*) is initiated in step 1, identifying the emergence of the sporadic application and scheduling the instances, including the sporadic application instance. Step 2 changes the *status* of the instances in the database of the sensor node that detected the event. In other words, the instances will be changed to *status* 2 (activated) or *status* 3 (deactivated). In step 3, it is sent the activated and deactivated *status* of the sensor node instances to the *sink*, through the DAO control message, disseminating information about the new scheduling of instances. Step 4 updates the database in *sink’s* memory, where the scheduling of the instances actually takes effect. These updates will be included in a DIO message in the next step (step 5), which is the transmission of the DIO message to synchronize all sensor nodes with the instances’ *status* changes and, consequently, the completion of the procedure of scheduling of the instances. Thus, the database of all sensor nodes and the *sink* are synchronized. Closing step 5 returns to step 1 to identify whether other sporadic applications have appeared or closed, and to carry out further scheduling.

### 4.5. Implementation Notes

We used Contiki (version 3.0) [28] embedded operating system to implement DYNASTI and Cooja simulator [27] to perform the simulations studies. There is no standardization of multiple instances yet, and, for the development of this work, we employed an adaptation of multiple instances implementation provided by [21,22]. Our implementation is available on GitHub [32].

Sporadic Event Detector is responsible to detect and report events that change instance scheduling. The event can be triggered by a motion, smoke sensor, etc., so Sporadic Event Detector is related to the application domain and needs to monitor the sensors in the node. Since we are using simulation, Sporadic Event Detector is implemented based in timers (as described in details in the next section), triggering events in some random time.

Instance Scheduler was implemented as a data structure that stores predefined information scheduling, i.e., sets of instances and their status (actived or deactivated). When an event is detected, Instance Scheduler selects a set accordingly and changes the instances’ status in the Instances Database, which is another data structure just to keep instances information.

We had to alter Contiki’s network stack in order to implement Message Controller and DIO/DAO messages modification. When an instance is deactivated, all its packets need to be dropped, so we added code in RPL to query the Instances Database before sending/receiving packets. In the same way, the control messages DIO and DAO were changed in order to forward the information from the Instances Database, thus DYNASTI uses the reserved field (reserved) of the RPL messages to transmit the status of the instances in a passive way. It is worth noting that DYNASTI takes advantage of constant send interval of these RPL messages (mostly controlled by the RPL trickle algorithm). Hence, DYNASTI does not incur new additional DIO and DAO messages.

All nodes have the described components excepting the sink, which does not detect events nor schedule instance. The role of the sink is just to disseminate the instance changes in the network, so that it does not have Sporadic Event Detector and Instance Scheduler.

## 5. Performance Evaluation and Results

This section presents the evaluation of our proposed DYNASTI solution. The evaluation was carried out by simulations to demonstrate the benefits of the proposal and the improvement of flexibility and dynamism levels when using multiple instances. Each result in the graphs is the averaging or summing of over 20 runs. The error bar was included in every graph using confidence interval based on confidence level of 95%.

We assessed (i) the overhead of the DIO and DAO in order to measure the impact of the control message interruption, (ii) the end-to-end delay for successfully received messages in the sink, assessing the impact they can have on different numbers of nodes and applications, (iii) the energy consumption which measures the total energy spent by the sensor nodes, and (iv) lost packets.

### 5.1. Network Configuration Environment

The network topologies are mostly based on spanning tree concept to allow nodes to be evenly distributed and to keep control of path length. Each node is included in all instances to provide more route options and thus making more complete use of alternative routes available on the network in order to meet the different requirements of each application. In addition, no one node is isolated from the network infrastructure, i.e., all nodes have at least one link with one or more adjacent nodes.

All the simulation’ parameters employed for the configurations of the sink and other nodes are shown in Table 2. They are based on the work provided by [21,22,33], where ZigBee Second Generation (IEEE 802.15.4) configurations are presented using CC2520 transceiver emulation.

### 5.2. Performance Metrics

As it was mentioned, the following performance metrics were used to evaluate the results: control message overhead (DIO and DAO), end-to-end delay of successfully received messages in the sink, energy consumed from the sensor nodes of the network, and lost packets. These metrics play a key role to assess the impact of the compared approaches on traffic performance and on the network itself.

DYNASTI aims to manage the multiple instances to better adapt the requirements of multiple applications by controlling the sent application messages, thus it is important to evaluate the delay and packet lost, mainly for critical applications. The packet lost number is the sum of the number of sent packets by nodes minus the number of received packets by sink (i.e., corrupted or dropped packets in the receiving queue). The end-to-end delay was calculated for critical applications and the application messages successfully received in sink. The delay is calculated separately for each critical application, the messages received by the sink were separated and then compared to the messages sent from the source. It was possible to filter the messages sent by an identifier located in the received messages. To reach this, the times of the received and sent messages were extracted and the difference between the receiving and sending times for each message was established, thus obtaining the delay per message. Equation (1) demonstrates the difference between the times of the message received and the message sent:

(1) MessageDelay=ReceivedMessage−SentMessage

Then, a sum of the messages delay was calculated, divided by the total number of messages successfully received by the sink (Equation (2)),

(2) $$\mathrm{Network}\;\mathrm{Delay}=\frac{\sum \mathrm{Message}\;\mathrm{Delay}}{\mathrm{Total}\;\mathrm{Received}\;\mathrm{Messages}}$$

DYNASTY also manages the control messages, activating or deactivating instances. Thus, the total number of the RPL control messages must be assessed. The total messages sent by all nodes in all active instances of the network, including the sink, is summed to analyze the overhead of the DIO and DAO control messages, obtaining the total control messages in the network.

Most of the smart devices of IoT LLNs are known to have very reduced battery life; therefore, it is mandatory to evaluate the impact of flexible and dynamic management of multiple instances on energy consumption. We have used the kinetic battery model implemented by Riker et al. [33] to calculate the consumed energy by the sensor nodes. When the simulation ends, the consumed energy from the sensor nodes is calculated, as showed in Equation (3):

(3) $$\mathrm{Averaged}\;\mathrm{Energy}\;\mathrm{Consumption}=\frac{\sum \;\mathrm{Node}\;\mathrm{Energy}\;\mathrm{Consumption}}{\mathrm{Number}\;\mathrm{of}\;\mathrm{Nodes}}$$

### 5.3. Application Scenario

For DYNASTI’s evaluation, we considered a Neighborhood Area Networks (NAN) with Advanced Metering Infraestructure (AMI) as real IoT scenarios [9,12,21,22] that use Smart Grid (SG) applications as well as enabling different types of applications. NAN enables power distribution with the ability of monitoring and controlling electricity delivery to each smart house; therefore, NAN assists the communication between Wide Area Network (WAN) and Home Area Network (HAN) in SG based mostly on the multi-hop communication [22]. Hence, a routing protocol is important in NANs with AMI. It is worth noting that our approach is not specific to AMI in SG, but it is mostly suitable to any context with multiple applications on the same topology with different QoS requirements. SGs are only an example of multiple applications scenario. Two scenarios of NAN with AMI are considered with two or three applications. This subsection describes information about each of the chosen applications and to which traffic class they belong.

Two types of traffic patterns are considered: regular and sporadic. Regular applications are running throughout the network lifetime and some of their requirements may change to meet the demand of other applications on the network. Sporadic applications will be executed when any random event arises (at least once during the simulation).

AMI is responsible for collection, analysis, and communication of energy consumption. It is capable of automatically and remotely measuring electricity usage, connecting and disconnecting a service, identifying and isolating interruptions, and monitoring voltage. The AMI modeling is based on the requirements of a smart grid NAN, which will contain the following applications: Application 1 (electricity monitoring), Application 2 (interruption identification), and Application 3 (voltage monitoring). The type of requirements and traffic pattern of these applications are: Application 1 is a regular and normal application, whereas Applications 2 and 3 are critical and sporadic applications.

The different priorities, the traffic pattern application, and the type and value of the requirement of each application are factors that influence the definition of scheduling. The sending of Application 1 messages is more constant on the network. Applications 2 or 3 sporadically arise (or both simultaneously) on the network; Application 1 must readjust its configuration parameters to release some of the traffic and resources; it was taking up on the network to communicate applications with more stringent network requirements.

Application 2 occurs sporadically on the network and also has a critical type requirement. It has more stringent requirements than Application 1 because it is responsible for triggering alerts if any interruption on the network is identified. Application 3 also occurs sporadically and is responsible for monitoring the variation in utility voltage (low or high voltage) because an equipment can be damaged. Therefore, Application 3 has more stringent requirements than the Applications 1 and 2.

### 5.4. Selection of the Scheduling of Instances

**Figure 7:**
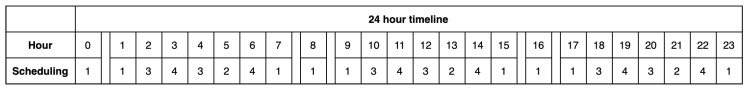
Example of timeline for the scheduling selection.

This section describes how the scheduling is selected in the simulation study. The total simulation duration is one day, with the time being divided into 24 slots (one slot per hour). A single scheduling of instances will be randomly selected for each hour using a uniform random distribution. Figure 7 shows the timeline with each scheduling instance for every hour.

The sporadic Applications 2 and 3 will run on the network for 20 min at maximum (duration time). This time is more than enough for the generated critical information by these applications reaches the sink. The initial minute of each sporadic application inside a single hour is randomly selected, since sporadic applications occur on the network eventually. The start minute of Application 2 is chosen according to an interval randomly between 0 and 20 min, using a uniform distribution. It is randomly chosen in an interval between 10 and 30 min for Application 3. These intervals were chosen so that Applications 2 and 3 are more likely to run concurrently when started on scheduling 4. Therefore, it is possible to evaluate the traffic performance when two sporadic applications coexist concomitantly.

### 5.5. Results

The multiple instance approaches described in Table 1 [21,22,23,24,25,26] have similar limitations, such as it employs a single instance per application, and there is neither instance management nor support to sporadic application, which results in static instances. For this reason, we developed a *Static Instance* approach that gathers all these limitations from the solutions in the related works.

The simulation aims to compare DYNASTI approach against to *Static Instances* approach. DYNASTI employs: scheduling of instances and management of the control and the application messages for each instance. *Static Instances* approach does not employ mechanisms of instance scheduling nor management of messages; however, they employ multiple instances, every application must be associated with a single instance which runs during the entire network lifetime in this approach.

#### 5.5.1. Varying the Number of Nodes

The impact between the DYNASTI solution and *Static Instances* is evaluated in the scenarios through network parameters when varying the number of nodes in the network, since the number of nodes will directly influence the number of sent control messages, end-to-end delay, energy consumption, and lost packets. We employed the 13, 25, and 37 as different scenarios of number of nodes (including the sink node, which is not evaluated). The node configurations were defined in Section 5.1, while the number of scheduling, instances, applications, and the random generation of scheduling are defined in Section 5.4. Figure 4, described in the previous section, provides an example of how the DYNASTI solution works on the scenarios; it explains how the scheduling of instances works.

DYNASTI and *Static Instances* have the same configurations, with two exceptions. First, *Static Instances* does not have sporadic applications. Second, in *Static Instances*, all applications send control and application messages throughout the total simulation time.

Table 3 presents the scheduling of the DYNASTI solution. Only Application 1 is active in Scheduling 1; therefore, it uses the entire network resource to transmit data. The *Message Interval* column specifies the application message interval in minutes—for instance, one message per node every five minutes. Unlike Scheduling 1, Scheduling 2 works differently: when Application 2 arises sporadically, the scheduler changes the instance of the Application 1 to adapt your traffic class and free up more resources. Hence, the message interval of Application 1 in Scheduling 2 (Instance 30) in the DYNASTI approach is one message for each 15 min. The time duration of Scheduling 2 is the same of Application 2 (i.e., 20 min) and, after its execution has ended, Instance 10 is reactivated. It is worth noting that the DYNASTI solution interrupts control and application messages when the sporadic application is not within 20 min of execution.

**Figure 8:**
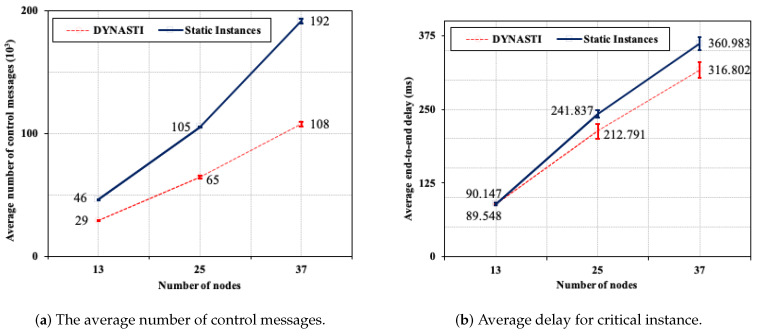
The average number of control messages and average delay for critical instance.

When comparing different number of nodes, Figure 8a shows that DYNASTI results in a much lower number of control messages for 25 (38.09%) and 37 (43.75%) scenarios than *Static Instances*. For instance, the number of control messages with 25 nodes in *Static Instances* is similar to the number of control messages with 37 nodes in DYNASTI. Figure 8b shows that the lowest end-to-end delay is achieved when DYNASTI is used; the difference between the two approaches increased as the number of nodes increased.

This greater reduction in the number of control and application messages, as well as the decreased delay with the DYNASTI solution can be explained by the efficiency of more flexible and dynamic management of multiple instances and applications, making it possible to interrupt instance control and application messages. This efficiency tends to improve with increasing node numbers for DYNASTI as more nodes result in more control and application messages as well as delay. Therefore, DYNASTI enables more scalability with respect to the number of network nodes.

**Figure 9:**
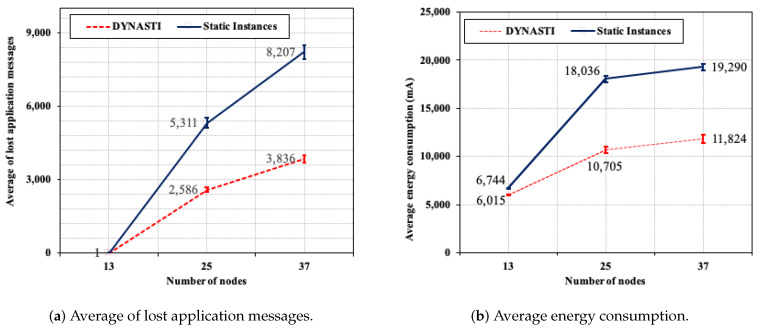
Average of lost application messages and average energy consumption.

Figure 9a compares the amount of lost application messages between the DYNASTI and *Static Instances*. The DYNASTI solution for all scenarios lost fewer messages than *Static Instances*; the difference was the highest in the 25 and 37 nodes’ scenarios, *Static Instances* has two times more losses than DYNASTI for these two scenarios. Figure 9b shows the power consumption for scenarios with 13, 25, and 37 nodes for the DYNASTI solution and *Static Instances*. The energy consumption pattern for both approaches is analogous, the difference is also significant, in which the highest energy consumption is achieved when *Static Instances* is used, i.e., *Static Instances* results in 68.48% (25 nodes) and 63.14% (37 nodes) more energy consumption than DYNASTI. Hence, the difference between approaches related to lost application messages and energy consumption increasing with the network size growth.

#### 5.5.2. Varying the Number of Applications

In this subsection, DYNASTI and *Static Instances* are evaluated with different quantities of application. Different numbers of applications are used to assess the approaches. Two scenarios were selected—the first with two applications and the second with three applications. To generate the obtained results, we employed the information from Table 2 and 25 nodes (one sink node and 24 sensor nodes).

It is possible to find four different types of scheduling of instances for the scenario with three applications of the DYNASTI solution. The possibilities and characteristics of each applications are described below. Two other possibilities of scheduling of instances emerged in the second scenario with two applications, when employed the DYNASTI solution.

Table 4 presents the scheduling for DYNASTI solution with three applications. Application 1 (electricity monitoring) is active on Scheduling 3, 4, 5, and 6. It uses all the necessary resources to transmit data on the network as long as it is active. Application 2 (interruption identification) is active in Scheduling 4 and 6. Application 3 (voltage monitoring) is active in Scheduling 5 and 6—all applications are active in Scheduling 6. Scheduling 4 differentiates from Scheduling 5 in the application message interval of the sporadic applications. The duration time of 20 min remains for both sporadic applications; however, these applications can be started at different times.

Table 5 shows the scheduling for the DYNASTI solution with two applications. Only Application 1 is active in Scheduling 7 and 8. Both applications are active in the Scheduling 8; Application 2 appears sporadically and lasts 20 min.

**Figure 10:**
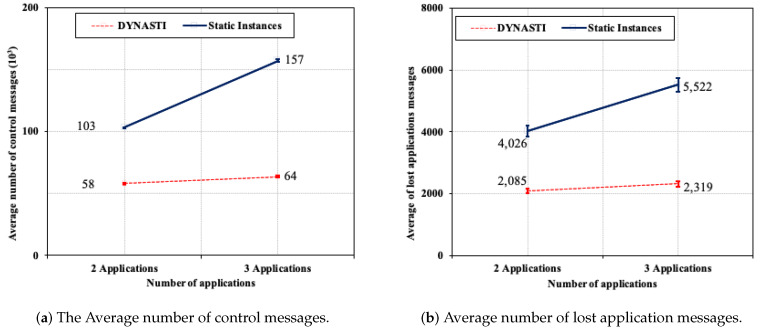
The average number of control messages and average number of lost application messages.

Figure 10a shows the average number of control messages for scenarios with two and three applications between the DYNASTI and *Static Instances* approaches. The control message transmissions occur throughout the simulation in *Static Instances* approach, thus the average number of control message is higher for both scenarios when *Static Instances* are employed. In the 2-application scenario, *Static Instances* result in an average of 77.58% more control messages than DYNASTI. In the 3-application scenario, *Static Instances* approach reach 143.31% more control messages than DYNASTI on average (more than double).

Hence, the total number of control messages increases much more when *Static Instances* approach are used as the number of applications increases. This is explained by the fact that the DYNASTI solution employs the message controller mechanism that makes it possible to stop control messages from instances of sporadic applications that do not need to be activated all the time.

Figure 10b shows a comparison between the average number of lost application messages. *Static Instances* approach results in a loss 93.09% more than the DYNASTI solution in the 2-application scenario. The *Static Instances* approach results in a lost application messages of 138.09% more than DYNASTI in the 3-application scenario (more than double). The message controller mechanism of the DYNASTI solution also assists with reducing the number of lost application messages, since there are much less control and application messages in the network than the *Static Instances* approach.

**Figure 11:**
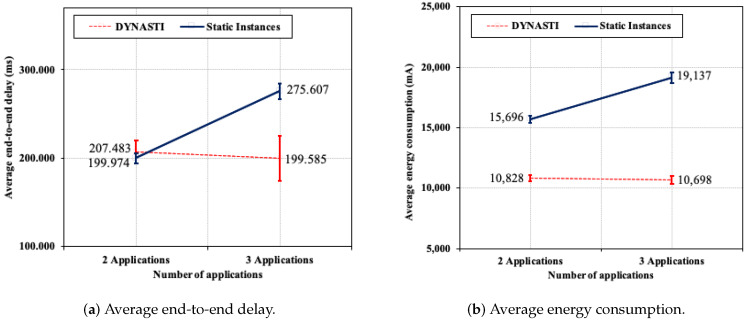
Average end-to-end delay and average energy consumption.

Figure 11a compares the delay of the DYNASTI solution with the *Static Instances* approach. Comparing the approaches in a scenario with three applications, there is a large difference between *Static Instances* and DYNASTI, approximately 76 ms. *Static Instances* increases the delay for critical application as the number of applications increases. Comparing the approaches in scenario with two applications, *Static Instances* achieves slightly shorter delay than DYNASTI, and the difference between them was approximately of 7 ms. This demonstrates that the DYNASTI solution was able to achieve the shortest delay with the highest number of applications because DYNASTI can achieve an optimized messages control of the multiple instances. Comparing the scenarios of two and three applications in *Static Instances*, it is possible to observe that there was a significant increase in the delay of approximately 76 ms as the number of applications increases, whereas DYNASTI reaches a very similar delay for both scenarios (two and three applications). This can be explained by the fact that DYNASTI interrupts control and application messages, while *Static Instances* does not.

Figure 11b shows the energy consumption for the DYNASTI and *Static Instances* approaches. In the scenario with two applications, a significant level of energy savings was achieved when using DYNASTI (31.01% more than *Static Instances*). In the scenario with three applications, this difference was bigger, with energy savings of 44.10% when using DYNASTI.

The results demonstrated that the DYNASTI solution enables dealing with more applications through the flexible management of multiple instances that allows for reducing the number of control messages, lost packets, and delay. In addition, DYNASTI is able to reduce power consumption, allowing longer device lifetimes.

#### 5.5.3. Varying the Application Message Send Intervals

The main goal of this section is to assess the utilization of adaptative application message send interval for a single application in the DYNASTI approach. The Instance Scheduler enables this adaptation interval in a dynamic way, since each application can have different instances. Each scheduled instance can employ a distinct application message send interval. To reach this, there are two evaluated DYNASTI approaches in this subsection. First, the DYNASTI approach which uses the Instance Scheduler and therefore it enables the dynamic and adaptive message send interval for a single application, called the *Adaptive Interval*. Second, the DYNASTI approach, which does not use the Instance Scheduler, is called *No Variation*. Thus, it is possible to evaluate the impact of the application message send interval, which is a configuration of the traffic class that aims to improve traffic differentiation by adapting the traffic of critical and normal applications in order to gather their requirements.

To achieve this purpose, the *Adaptive Interval* approach appropriately uses two scenarios with two different intervals for each. In the first scenario, *Adaptive Interval* uses two instances associated with the regular application with the intervals of 5 and 7 min and an instance for sporadic application with three-minute intervals. For the second scenario, *Adaptive Interval* uses two instances associated with the regular application with the intervals 5 and 15 min and the same interval for sporadic application (three minutes).

In total, there are three instances in each *Adaptive Interval* scenario, but only two run concurrently. When sporadic application appears, the regular instance with shorter message interval (five minutes) is stopped. The second instance of the regular application is then activated, with a higher application message sending interval (7 or 15 min), now representing the regular application.

In addition, there is the *No Variation* approach, which makes use of a single instance for regular application and one instance for sporadic application. In this approach, when the sporadic application comes up, the regular application instance continues to perform with the same traffic class, without adaptation to the regular application.

Simulations tests were performed using the information from Table 2 to assess this attribute of the traffic class (send interval). Two applications were used: electricity monitoring, for regular application and normal requirement, and interruption identification, for sporadic application and critical requirement (as described in Section 5.3).

Table 6 shows the scheduling for the *Adaptive Interval* approach, using one message per node in each 7 or 15 min as interval variation. When the scheduling is changed, Application 2 (interruption identification) starts running, Application 1 (electricity monitoring) has its instance changed and now it uses Instance 30, which has a greater application message send interval (one message in each 7 or 15 min). Thus, this switching between the two instances of Application 1 makes it possible to prioritize the traffic of the Application 2, as Application 1 decreases network traffic and therefore frees more resources to sporadic application. The duration time of Application 2 is 20 min.

Table 7 shows scheduling for the *No Variation* approach using one message per node in every five minutes as the application message send interval. Only application 1 (electric current monitoring) is active in scheduling 11 and 12. Both applications are active in scheduling 12.

**Figure 12:**
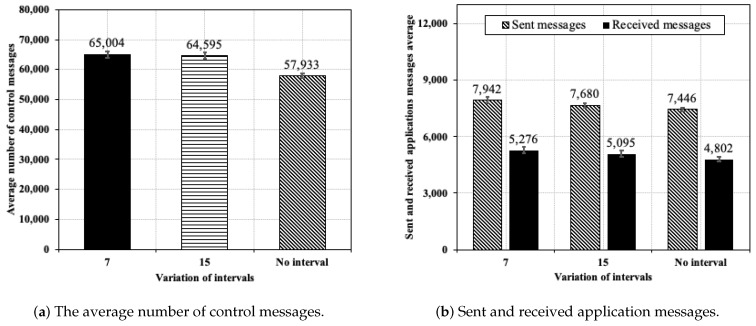
The average number of control messages and sent and received application messages.

Figure 12a presents the achieved results in the average total control messages for *Adaptive Interval* and *No Variation* approaches. The average of sum of the total control messages (DIO and DAO) in all simulations was used to calculate the results. There was a slight difference between the two interval variations of the *Adaptive Interval* approach.

The number of control messages for the *Adaptive Interval* approach increase, yielding about 11.49% more control messages than the *No Variation* approach. This behavior is due to a couple of reasons—first, the fact that, in *Adaptive Interval* approach, there are three instances, while, in the *No Variation* approach, there are only two instances. Second, it is important to note that the management of control messages for instances 10 and 30 of the same regular application are effected by changing the *status* of these instances, and these *status* need to be propagated on the network. Therefore, the scheduling change does not occur immediately on all nodes and, consequently, the control messages from both instances are being transmitted for a period of time. Still, the increase of the number of control messages (about 11.49%) was not proportional to the number of instances in the network (33.3%) due to the more optimized management provided by DYNASTI.

Figure 12b is used to compare results between sent and received application message averages. The *Adaptive Interval* approach sends and receives more application messages than *No Variation* approach for both variations; the variation of 7 min sends more 6.7% and receives more 9.87% and the variation of 15 min sends more 3.14% and receives more 6.10% than a *No Variation* approach. The slight and higher number of application messages sent and received is justified by the same reason as the increase in the control messages, i.e., due to the time taken for the RPL control messages to propagate and to stop instance 10 when the sporadic application arises. In addition, while the variations of *Adaptive Interval* approach result in 2666 and 2585 lost messages for the 7 min and 15-minute intervals, respectively, the *No Variation* approach results in 2644 lost messages. These results show that the smallest number of lost messages was achieved when using the greatest application message interval (15 min), which may be justified by freeing resources from the regular application instance that employs this greater interval, favoring messages of the critical application though a smaller loss.

Hence, it is worth noting that the 15-minute variation lost fewer messages and could provide more QoS for your critical application; thus, critical application receives more packets and generally loses fewer application messages than the *Adaptive Interval* approach with a 7-minute variation and the *No Variation* approach.

**Figure 13:**
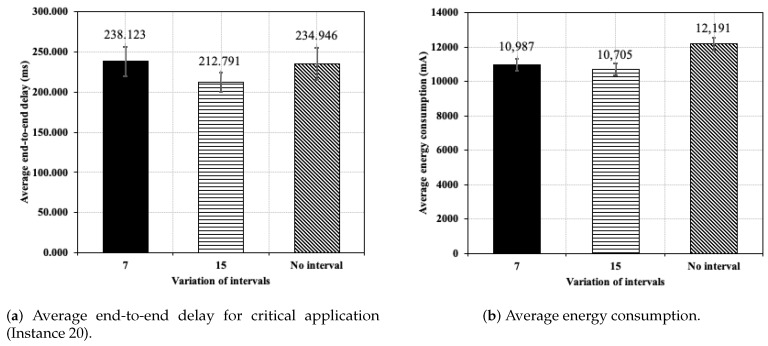
Average end-to-end delay for critical application (Instance 20) and average energy consumption.

A comparison is carried out between the average delay for *Adaptive Interval* and *No Variation* approaches, as it is shown in Figure 13a. In this case, only messages successfully received in sink are considered. The results demonstrate that the *Adaptive Interval* approach using seven minutes and *No Variation* approaches resulted in very similar values, since the difference was very small, only 3.18 ms. However, the difference between the 15 min *Adaptive Interval* approach and the *No Variation* approach was 22.155 ms, about a 10% difference. The higher the number of application messages, the greater the required time to transmit the messages and, therefore, higher the delay. Hence, the *Adaptive Interval* approach using 15 min sends a smaller number of messages for the regular application; thus, this variation results in shorter delay than the *No Variation* approach. In this sense, the use of different message send intervals helped to improve service differentiation for the critical application, prioritizing more the traffic of this application.

The average energy consumed of all network nodes can be seen in Figure 13b, except for sink. Again, there was a slight difference between the two interval variations of the *Adaptive Interval* approach. The *Adaptive Interval* approach surprisingly reached lower power consumption than the *No Variation* approach, which is 9.87% (1204.82 mA) and 12.18% (1486.78 mA) lower in 7 min and 15 min, respectively. It is worth pointing out that the Instance 30 has a higher application message sending interval (7 min and 15 min) than Instance 10. Therefore, the nodes’ radios remain in *sleep mode* for a longer amount of time in *Adaptive Interval* approaches. Although with the greater amount of control and application messages sent and received with the *Adaptive Interval* approach, power consumption is significantly lower in this mode [34], which enabled a reduction in power consumption.

Despite the slight difference between the message sending intervals between instances 10 and 30 (2 and 10 min, respectively), promising results can be seen from the use of different instances for the same application using a different send interval for each instance of the same application. For example, a greater reduction in energy consumption was achieved even though there were more control and application messages. A reduction in delay of the critical application showed that using separate intervals for the same regular application can help to improve delay performance, thus providing service differentiation for more critical applications.

#### 5.5.4. Results Summary

The results showed that the DYNASTI reaches significant improvement in traffic performance for non-critical and critical applications as well as coexistence between regular and sporadic applications. DYNASTI also shows itself as a more scalable solution for a greater number of nodes and applications. Furthermore, it achieves better service differentiation for critical applications.

## 6. Conclusions and Future Work

The development of IoT based on LLNs motivated the scientific research work to improve routing through multiple instances. It opened the possibility of bringing the context of multiple applications in IoT with the feasibility of multiple instances and evolving the state of the art with new solutions that consider these devices with limited resources. Related works present multi-instance usage assessments; however, they do not take full advantage of multiple instances towards multiple application scenarios.

DYNASTI is a solution of multiple instances towards multiple applications, which aims to bring a more flexible and dynamic management of multiple instances, enables the suitability of normal applications while critical applications have priorities in traffic, and coexistence between regular and sporadic applications. In addition, DYNASTI enables coexistence between multiple instances and single/multiple applications competing for available network resources while taking into account each application’s set of requirements. Hence, the DYNASTI solution achieves service differentiation by combining all developed mechanisms, such as managing the sending of control and application messages of instances (message controller), scheduling of instances (instance scheduler), using different messaging intervals and, routing metrics, which makes applications more flexible and can free up resources and reduce network overhead.

The results obtained by DYNASTI showed an overall efficiency improvement for the network, since there was an enhanced optimization of resources, mainly in the reduction of the control messages and energy consumption. DYNASTI results in fewer lost applications’ messages and shortest delay for critical applications compared to the state-of-the-art approach in which there is no interruption of control and application messages and scheduling of instances. For future work, it is desirable to run the DYNASTI solution on a real prototype to assess its results with real sensor devices as well as to apply machine learning techniques to instance scheduling.

## Figures and Tables

**Table 1:** Comparison between the related work against our proposal.

Approach	Metric	Number of Instances Per Application	Differentiation of Services for Applications	Traffic Pattern
Long et al. [23]	ETX	1	No	Regular
Rajalingham et al. [24]	ETX e HC	1	No	Regular
Banh et al. [25]	ETX e HC	1	No	Regular
Barcelo et al. [26]	ETX, PDR e HC	1	Yes	Regular
Nassar et al. [21,22]	*mOFQS*	1	Yes	Regular
DYNASTI	*Multiple Metrics*	multiple	Yes	Regular and Sporadic

**Table 2:** RPL parameters and nodes’ configuration on Cooja simulator.

Parameters	Values
Initial Residual Energy	80.000 mA
Routing Metric	ETX
Control Messages Length (DIO e DAO)	4 bytes
Application Message Payload	40 bytes
Sensor Actuator	WiSMote
Simulation Time	24 h
PHY layer	IEEE 802.15.4
MAC layer	ContikiMac
Microcontroler	MSP430
Transceiver	CC2520
RAM memory	768 bytes
Processor Clock	1–16 MHz
Maximum transmission rate	250 kbps

**Table 3:** Scheduling for DYNASTI in scenarios varying the number of nodes.

Application	Instance	Traffic Pattern	Traffic Type	Messages Interval	Scheduling
1	10	Regular	Normal	5 min	1
1	30	Regular	Normal	15 min	2
2	20	Sporadic	Critical	3 min	2

**Table 4:** Scheduling of DYNASTI with three applications.

Application	Instance	Traffic Pattern	Traffic Type	Messages Interval	Scheduling
1	10	Regular	Normal	7	3,4,5,6
2	20	Sporadic	Critical	5	4,6
3	30	Sporadic	Critical	3	5,6

**Table 5:** Scheduling of DYNASTI with two applications.

Application	Instance	Traffic Pattern	Traffic Type	Messages Interval	Scheduling
1	10	Regular	Normal	7	7,8
2	20	Sporadic	Critical	3	8

**Table 6:** Scheduling for DYNASTI for the *Adaptive Interval* approach.

Application	Instance	Traffic Pattern	Traffic Type	Messages Interval	Scheduling
1	10	Regular	Normal	5	9
1	30	Regular	Normal	7 or 15	10
2	20	Sporadic	Critical	3	10

**Table 7:** Scheduling for DYNASTI for the *No Variation* approach.

Application	Instance	Traffic Pattern	Traffic Type	Messages Interval	Scheduling
1	10	Regular	Normal	5	11, 12
2	20	Sporadic	Critical	3	12

## References

[1] Hammi B., Khatoun R., Zeadally S., Fayad A., Khoukhi L. (2018). IoT technologies for smart cities. IET Netw..

[2] Alsaig A., Alagar V., Chammaa Z., Shiri N. (2019). Characterization and Efficient Management of Big Data in IoT-Driven Smart City Development. Sensors.

[3] Al-Fuqaha A., Guizani M., Mohammadi M., Aledhari M., Ayyash M. (2015). Internet of Things: A Survey on Enabling Technologies, Protocols, and Applications. IEEE Commun. Surv. Tutor..

[4] Amah T.E., Kamat M., Bakar K.A., Moreira W., Oliveira A., Batista M.A. (2020). Preparing opportunistic networks for smart cities: Collecting sensed data with minimal knowledge. J. Parallel Distrib. Comput..

[5] Vejlgaard B., Lauridsen M., Nguyen H., Kovacs I.Z., Mogensen P., Sorensen M. Coverage and Capacity Analysis of Sigfox, LoRa, GPRS, and NB-IoT. Proceedings of the 2017 IEEE 85th Vehicular Technology Conference (VTC Spring).

[6] Ratasuk R., Vejlgaard B., Mangalvedhe N., Ghosh A. NB-IoT system for M2M communication. Proceedings of the 2016 IEEE Wireless Communications and Networking Conference.

[7] Latre S., Leroux P., Coenen T., Braem B., Ballon P., Demeester P. City of things: An integrated and multi-technology testbed for IoT smart city experiments. Proceedings of the 2016 IEEE International Smart Cities Conference (ISC2).

[8] Pasetti M., Sisinni E., Ferrari P., Rinaldi S., Depari A., Bellagente P., Della Giustina D., Flammini A. (2020). Evaluation of the Use of Class B LoRaWAN for the Coordination of Distributed Interface Protection Systems in Smart Grids. J. Sens. Actuator Netw..

[9] Kuzlu M., Pipattanasomporn M., Rahman S. (2014). Communication network requirements for major smart grid applications in HAN, NAN and WAN. Comput. Netw..

[10] Cam-Winget N., Hui J., Popa D. (2017). Applicability Statement for the Routing Protocol for Low-Power and Lossy Networks (RPL) in Advanced Metering Infrastructure (AMI) Networks.

[11] Khanna A., Anand R. IoT based smart parking system. Proceedings of the 2016 International Conference on Internet of Things and Applications (IOTA).

[12] Xu S., Qian Y., Hu R.Q. (2015). On Reliability of Smart Grid Neighborhood Area Networks. IEEE Access.

[13] Oliveira-Jr A., Resende C., Gonçalves J., Soares F., Moreira W. IoT Sensing Platform for e-Agriculture in Africa. Proceedings of the 2020 IST-Africa Week Conference (IST-Africa).

[14] Davito B., Tai H., Uhlaner R. (2010). The smart grid and the promise of demand-side management. Mckinsey Smart Grid.

[15] Budka K.C., Deshpande J.G., Doumi T.L., Madden M., Mew T. (2010). Communication network architecture and design principles for smart grids. Bell Labs Tech. J..

[16] Taxonomy B.S.G. (2015). A System View from a Grid Operator’s Perspective. http://www.bkw.ch/fileadmin/user_upload/3_Gemeinden_EVU/gem_smart_grid_systematik_en.pdf.

[17] Delphinanto A., Koonen T., den Hartog F. End-to-end available bandwidth probing in heterogeneous IP home networks. Proceedings of the 2011 IEEE Consumer Communications and Networking Conference (CCNC).

[18] Sobral J.V.V., Rodrigues J.J.P.C., Rabêlo R.A.L., Al-Muhtadi J., Korotaev V. (2019). Routing Protocols for Low Power and Lossy Networks in Internet of Things Applications. Sensors.

[19] Watteyne T., Winter T., Barthel D., Dohler M. (2009). Routing Requirements for Urban Low-Power and Lossy Networks.

[20] Alexander R., Brandt A., Vasseur J., Hui J., Pister K., Thubert P., Levis P., Struik R., Kelsey R., Winter T. (2012). RPL: IPv6 Routing Protocol for Low-Power and Lossy Networks.

[21] Nassar J., Gouvy N., Mitton N. Towards Multi-instances QoS Efficient RPL for Smart Grids. Proceedings of the 14th ACM Symposium on Performance Evaluation of Wireless Ad Hoc, Sensor, & Ubiquitous Networks—PE-WASUN ’17.

[22] Nassar J., Berthomé M., Dubrulle J., Gouvy N., Mitton N., Quoitin B. (2018). Multiple Instances QoS Routing in RPL: Application to Smart Grids. Sensors.

[23] Long N.T., Uwase M.P., Tiberghien J., Steenhaut K. QoS-aware cross-layer mechanism for multiple instances RPL. Proceedings of the 2013 International Conference on Advanced Technologies for Communications (ATC 2013).

[24] Rajalingham G., Gao Y., Ho Q.D., Le-Ngoc T. Quality of service differentiation for smart grid neighbor area networks through multiple RPL instances. Proceedings of the 10th ACM Symposium on QoS and Security for Wireless and Mobile Networks—Q2SWinet’ 14.

[25] Banh M., Mac H., Nguyen N., Phung K.H., Thanh N.H., Steenhaut K. Performance evaluation of multiple RPL routing tree instances for Internet of Things applications. Proceedings of the 2015 International Conference on Advanced Technologies for Communications (ATC).

[26] Barcelo M., Correa A., Vicario J.L., Morell A. (2016). Cooperative interaction among multiple RPL instances in wireless sensor networks. Comput. Commun..

[27] Sehgal A. (2013). Using the Contiki Cooja Simulator.

[28] Österlind F. (2006). A Sensor Network Simulator for the Contiki OS.

[29] Winter T., Thubert A.B.P., Clausen T., Hui J., Kelsey R., Levis P., Pister K., Struik R., Vasseur J. (2012). RPL: IPv6 Routing Protocol for Low Power and Lossy Networks, (RFC 6550).

[30] De Couto D.S., Aguayo D., Bicket J., Morris R. A high-throughput path metric for multi-hop wireless routing. Proceedings of the 9th Annual International Conference on Mobile Computing and Networking.

[31] Thubert P. (2012). Objective Function Zero for the Routing Protocol for Low-Power and Lossy Networks (RPL).

[32] Junior S.A. (2020). DYNASTI Implementation. Github. https://github.com/SidneiJuniorx/contiki-dynasti.

[33] Riker A., Curado M., Monteiro E. Neutral Operation of the Minimum Energy Node in energy-harvesting environments. Proceedings of the 2017 IEEE Symposium on Computers and Communications (ISCC).

[34] Varghese B., John N.E., Sreelal S., Gopal K. (2016). Design and development of an RF energy harvesting wireless sensor node (EH-WSN) for aerospace applications. Procedia Comput. Sci..

